# Challenges in Implementation of Mother Milk Banks in Rajasthan: a Situational Analysis

**DOI:** 10.34763/jmotherandchild.20212502.d-21-00009

**Published:** 2022-04-01

**Authors:** Neha Mantri, Akhil D Goel, Nitin K Joshi, Pankaj Bhardwaj, Vaishali Gautam, Manoj K Gupta

**Affiliations:** 1 School of Public Health, All India Institute of Medical Sciences, Jodhpur, Rajasthan, India.; 2 Community Medicine and Family Medicine, All India Institute of Medical Sciences, Jodhpur, Rajasthan, India.; 3 Community Medicine and Family Medicine, Demonstrator School of Public Health, All India Institute of Medical Sciences, Jodhpur, Rajasthan, India.; 4 Community Medicine and Family Medicine, Coordinator School of Public Health, All India Institute of Medical Sciences, Jodhpur, Rajasthan, India.

**Keywords:** Breastfeeding, breastmilk, milk banks, lactation, root cause analysis, infants

## Abstract

**Background:**

Breastmilk is the baby’s “first vaccine”. Donated human milk (DHM) is the next best alternative when a mother’s milk is not available, as recommended by WHO- UNICEF. DHM as a nutritional source provides similar immune protection and may prove revolutionary in reducing neonatal morbidity and mortality. The study aimed to explore the challenges in the implementation of selected Mother Milk Banks (MMBs) of Rajasthan.

**Material and methods:**

A qualitative in-depth interview of key stakeholders was conducted to know the various facets of the challenges in milk banking practices using the Root Cause Analysis framework.

**Results:**

The system challenges identified in the functioning of AMMBs were lack of recurring funds, dedicated lactational counselors, and trained technicians. Databases for demand-supply estimates were also lacking. The community challenges were low acceptance of DHM due to safety concerns, risk of disease transmission, and quality of donated milk. Moreover, the religious stigma and cultural beliefs regarding the transfer of heredity traits and decrease in mother-child affection act as barriers in donating milk.

**Conclusion:**

For acceptance and availability of DHM, Social Behavior Communication Change (SBCC) interventions must be incorporated early during the antenatal check-up period. Our study highlighted the role of education; motivation by healthcare providers has a major influence on infant feeding choices. In a developing country such as India, where the frameworks concerning the development of mother milk banks are still maturing, our study findings provide baseline information to address the barriers in the implementation of mother milk banks in India.

## Introduction

***Breastfeeding*** is an innate right of every newborn and a complete source of nutrition for newborns. The mother must introduce breastmilk to the infant at the earliest opportunity. A child must be exclusively breastfed till 6 months, after which supplementary food can be added [1]. Breastmilk is an astonishing emulsion that contains nutrients, enzymes, immune factors, and growth hormones for the adequate growth of a baby [2]. Together with this, the immune-protective and digestion factors make it supreme among all other feeding competitors. Omega-3 fatty acids are essential for brain development, and are supplied only in breastmilk [3]. For the baby, it has all the nutrients in proper proportion for optimal growth and development [3]. For the mother, it reduces the probabilities of cancer, delays subsequent pregnancy, promotes early expulsion of the placenta, and reduces post-pregnancy complications.[3] Above all, it ensures a strong mother-child bond [3].

***Donated human milk (DHM)*** is the desirable alternative when a mother’s milk is not available; this was communicated as a joint statement by WHO - UNICEF through the Baby-Friendly Hospital Initiative in 1980 [4]. Donor human milk provides similar immune protection and may prove revolutionary in reducing infant morbidity and mortality. It becomes the responsibility of the government to achieve breastfeeding goals such as promotion of exclusive breastfeeding, reduction in the use of formula feed, establishing milk banks, and more.

A ***Mother Milk Bank*** (***MMB***) is a facility that allows the collection and distribution of human milk donated by lactating mothers other than the biological mother. The last few decades have seen the establishment of several milk banks as a nutritional source for sick and low-birth-weight infants, orphan and abandoned children, medical conditions where a mother cannot produce sufficient milk for the infant, and unfortunate maternal death.

Despite the worldwide decline in child mortality, the infant mortality rate in India continues to be over 30 per 1000 live births [3]. Malnutrition in children is more an interplay of female illiteracy, ignorance about the nutritional needs of infants and young children, and poor access to health care. Besides these factors, 27 million babies are born per annum in India, of which 3.5 million are preterm and 7.5 million are low birth weight [5]. These babies are vulnerable in terms of survival and cognitive development and typically have feeding problems due to their medical conditions.

***In Asia***, the first milk bank, “Sneha”, was established under the leadership of Dr. Armeda Fernandez at Sion hospital in Dharavi, Mumbai, in 1989; this is now a 30-year-old body nurturing the newer establishments through its experience [6]. There are more than 50 donor milk banks in India to date. In 2014, human milk banking guidelines were published which became the basis for the establishment of new MMB [7]. Later, standard operating protocol (SOP) for human milk collection, storing, and dispensing for babies admitted at health facilities was developed, and manuals were written by the Government of India (GOI) in 2017 [5]. DHM offers immense potential to curb the high rates of infant mortality, morbidity, and malnutrition [5]. Despite the established benefits of DHM, its growth in India is limited.

***Rajasthan Government*** launched “***Amrut-Kaksh*”** as a component of the National Nutrition Mission 2022, to communicate with rural communities to promote exclusive breastfeeding till 6 months [8]. The first mother milk bank, “Jeevan Dhara”, was inaugurated in 2015 in Udaipur by an NGO in the government setup. Also, “Divya Mother Milk Bank”, the first community milk bank, was established as a non-public setup in Udaipur [9]. Today, Rajasthan has around 19 milk banks, under the name ***Aanchal Mother Milk Banks (AMMB)***, which are the highest in number as compared to other states. More milk banks are in the pipeline [8].

The present study discovers the challenges within the operationality of the established Aanchal Mother Milk Bank in Rajasthan. It also captured the perception of beneficiaries and health care providers for the utilisation of donor milk. This, in turn, would offer evidence for the successful implementation of lactation management centers at public health facilities, and would benefit the most vulnerable part of the community, that is, infants, by reducing mortality rates.

## Material and methods

This qualitative study was conducted to explore the challenges faced in the implementation of the AMMBs in Rajasthan. For study purposes, data was collected from the currently functional three oldest AAMBs, located at Tonk, Alwar, and Chittorgarh district of the Rajasthan.

Stakeholders related to the functioning of selected AMMBs are the medical officer, mother milk bank in-charge, staff nurse, technician, lactation counselor, and administrative officer. Lactating women visiting the selected mother milk banks were selected as participant mothers.

*Criteria for Donors*: A lactating woman who is in good health, feeds her baby satisfactorily, is willing to undergo blood testing, and has the enthusiasm to become a breastmilk donor. A donor is disqualified if she fails the checklist for becoming a donor, which includes viral blood markers, alcohol/tobacco use, undergoing any radiotherapy, mastitis, or any fungal infection in the breast area [7].

*Criteria for Recipients*: DHM is supplied on a priority basis for preterm and sick babies, mothers with postpartum illness, mothers with insufficient/absent lactation. If supplies of DHM are sufficient, they may be given when conditions such as the temporary interruption of breastfeeding, as when certain breast conditions (breast engorgement, cracked nipples) reduce the required feed of the infant. DHM is also made available when alternative feeding is required: for abandoned neonates, in case of maternal death, adopted neonates, a child unable to suck, child potential for multiple co-morbidities, or newborn weight loss [7].

A situational analysis was planned to explore the functioning of selected AMMBs after permission from concerned authorities. A face-to-face interview was conducted using the In-depth interview (IDI) guide for lactating mothers (n=30) and health care providers (n=25) to understand their knowledge, attitudes, and practices on DHM and MMB. The questionnaire was also directed towards eliciting information on sociodemographic details. Interviews were conducted in local languages – Hindi and Marwari – ensuring respect for local cultural and religious sensitivities. The participant was informed about the aim of the study and informed consent was obtained. Those who refused consent were excluded from the study (n=5). Privacy, confidentiality, and volunteerism of participants were ensured. Interviews lasted for 25–30 minutes. Audio-recording was done to collect data and field notes were made. After content analysis of interview data, transcripts were obtained. After comparing differences and similarities, codes were formed by two authors. The process of writing and rewriting themes continued until participants’ expressions were captured precisely. Data saturation was achieved till no new themes emerged. After discussion with experts, themes that best expressed participants' experiences were finalised. Verbatims of the lactating mothers are symbolised as M and the healthcare providers as H. Interviews were conducted by one researcher to ensure a high level of consistency [10].

The Root Cause Analysis framework was used to understand the various challenges. This is a comprehensive approach to discover the root causes of problems or challenges. A fishbone diagram (Ishikawa model) was used to summarise the underlying bottlenecks to find appropriate solutions [11].

## Results

It was insightful to assess the current practices and actual performance of AMMBs. A total of 30 lactating mothers and 25 health care providers were interviewed in 3 selected AMMBs. Most mothers belong to the age group 20–34 years and were unemployed. The study shows 66.6% have a residence in urban areas, and 40% were illiterate. Most of the participating health care providers were 25–36 years old and have a residence in urban areas (92%). Around 66.6% of mothers had heard about MMB through health care providers. The remaining 33.3% of mothers had heard from relatives who had used the service of AMMB. Few mothers (30%) fear donating milk due to less milk for their babies, but on counseling, they agreed. Few mothers (23.3%) were unwilling to utilise donated milk due to fear of infection.

## Challenges in Aanchal Mother Milk Banking Practices

**Figure 1:**
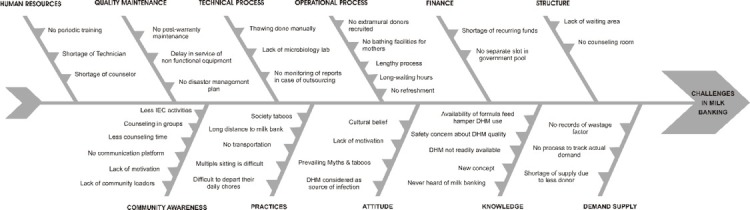
The challenges observed in the functioning of the Aanchal mother milk banking practices.

The challenges observed in the functioning of the Aanchal mother milk banking practices are classified into major themes as system and community challenges **(Fig 1)**. The system challenges have been further described using subthemes such as structure, financial support, operational process, technical procedure, quality maintenance, human resources, demand-supply ratio, knowledge, attitude, and practices about DHM and milk banking. The community challenges are categorised as lack of awareness and motivation, cultural myths, and taboos. The verbatim quotes of the participants are discussed in **Supplementary file 1**.

## A System challenges

### 1 Structure

(**Table 1**) This matrix elaborates the level-based Facility Lactation Management Centers at a three-tier healthcare delivery system. A high-complexity to a less complex model of lactation management centers is established according to the need for health care facilities. The design of the apex unit, the Comprehensive Lactation Management Center (CLMC), is in line with its purpose of collecting, screening, processing, storing, and distributing milk established at medical college/district hospitals. This is followed by a Lactation Management Unit (LMU) setup at subdistrict hospitals with the aim of milk expression, storage, and dispensing. Lactation Support Units (LSU) are delivery points established to maintain a continuum of care, even in remote areas.

**Table 1:** Matrix Elaborating Levels of Facility-Based Lactation Management Centers in Public Healthcare System

	CLMC*	LMC†	LSU‡
**Other Name**	Tertiary Milk Bank	Mini Milk Bank	Milk Storage Unit
**Infrastructure**	Elaborate setup for heterologous milk collection, screening, processing, storing and distributing DHM^§^.	Setup with homologous milk collection, storage and dispensing of milk for consumption by her baby.	Setup to store small quantity of processed milk on a consumption basis.
**Location**	Medical College with NICU^¶^ or Large District Hospital	District Hospital /Subdistrict Hospital /First Referral Units (FRUs) with functional SNCU^#^	At Subdistrict Hospital /CHC/PHC with high case load NBSU**. (all delivery points)
**Complexity and Resource Intensity**	Highest	High	Low
**Responsibility**	Act as an ancillary support to the Baby Friendly Hospital Initiative (BFHI). Act as a supply unit for Milk Storage Unit.	Provide lactation support to all mothers within facility.	To maintain continuum of care by providing round the clock breastfeeding support, lactational counselling and Kangaroo Mother Care (KMC) support to mothers.
**Recommended Space**	Approximately 350 square meters	Approximately 160 square meters	Any identified space for breastfeeding as per Mother Absolute Affection (MAA) guidelines.
**Recommended Minimum Bedded**	20 bedded NICU along with SNCU	12 bedded SNCU	High caseloads NBSU
**Recommended Full-time Staff**	Manager (1) Lactation Support Staff (5) (only female staff) Technician (1)	Lactation Support Staff (2) (only female staff)	No dedicated human resource.
**Recommended Part-time Staff**	Microbiologist (1) Hygiene Helper (one per shift)	Hygiene Helper (1)	
**Recommended Existing Staff in facility to be utilized**	Neonatologist (2)	Neonatologist (2)	FIMNCI Nurse†† and Doctor in-charge NBSU
**Set-up cost (One time)**	Rs. 33,114,00/-	Rs.9,75,000/-	Cost of deep freezer, foot-operated basin, glass bottles if not already available.
**Recurring cost Per annum**	Rs. 14,76,000/-	Rs.4,65,000/-	-

*CLMC-Comprehensive Lactation Management Center †LMC-Lactation Management Center ‡LSU-Lactation Supply Unit §DHM-Donor Human Milk ¶ NICU-Neonatal Intensive Care Unit #SNCU- Sick Newborn Care Unit **NBSU- New Born Stabilisation Unit ††FIMNCI- Facility based Integrated Management of Neonatal and Childhood Illness. *Reference: National Guidelines on Lactation Management Centers in Public Health Facilities, Child Health Division, Ministry of Health and Family Welfare, Government of India (2017)*

All AMMBs were functioning in close vicinity to the Neonatal Intensive Care Unit (NICU). All of them strictly follow National Lactation Management Center guidelines (Ministry of Health & Family Welfare, GOI, 2017) together with standard operating protocols [7]. The infrastructure was well constructed and maintained but there was a constraint on waiting areas and space in counseling rooms.

### 2 Financial Support

The most pressing concern revealed from all AMMBs was the dearth of recurring operating funds. There is no designated fund for AMMBs in the government pool and hence they are linked into the district fund. Thus, there is a requirement for streamlining financial mechanisms for the smooth running of AMMBs.

### 3 Operational Process

All potential donors are systematically screened and the process of milk collection follows strict hygiene protocols. No donors from outside the hospital facility are recruited in AMMB. As per the recommendation, the availability of bathing facilities for donor mothers will help in reducing the contamination of milk. However, the observed facilities were still in the process of developing such facilities for the donor mothers. The pooling of milk from multiple mothers was done and the system was developed to track aliquots. Records are maintained in both manual and electronic formats. Preterm and term milk is stored separately in one of the AMMBs.

### 4 Technical procedure

Most of the AMMBs were well equipped with electric breast pumps for milk expression, refrigerators, deep-freezer, and shaker water bath with equipment manual. But there have been issues in the annual maintenance contracts for equipment. Also, sometimes nonfunctional equipment disrupts the milk bank processing. All AMMBs employ the Holder pasteurization method followed by a water shaker bath. Thawing is done manually, which is cumbersome and a source of contamination.

### 5 Quality maintenance

There is a lack of post-warranty maintenance for equipment. Many times, due to lack of manpower or reagents, lab cultures are outsourced. There is a need to develop a mechanism to monitor such outsourced tests. A disaster management plan was lacking in all the AMMBs. Pest control measures were strictly followed.

### 6 Human Resources

All AMMBs have reported a shortage of human resources, especially technicians and lactation counselors. Most healthcare providers find it difficult to manage with a scarcity of adequate human resources. Less time was dedicated to motivating mothers due to a shortage of human resources.

### 7 Demand-supply ratio

Nursery keeps an advanced stock of DHM for feeding babies, especially during night hours. But the systematic records are lacking in terms of actual demand and supply daily. Sometimes, the recruitment of inadequate donors causes a supply shortage in NICU. Some mothers are in hope of getting continuous supply through a milk bank after they get discharged.

### 8 Knowledge about DHM and Mother Milk Banking

Most healthcare providers stated that when a newborn is unable to suck or when mother’s milk is not available due to medical conditions, they promote DHM as the alternate feeding option. All healthcare providers were of the opinion that donated human milk is superior to formula feeding, and that it also helps sick newborns to recover faster. Most of the mothers agreed that they heard about the mother milk banking concept in this hospital by staff. Before, they knew the concept of milk sharing among blood relatives. Most mothers expressed positive views on donating or sharing milk for needy sick newborns but lack proper knowledge about the process of milk banking.

### 9 Attitude towards DHM and Mother Milk Banking

- ***Willingness for Breastmilk Donation and Utilisation***

Most of the healthcare providers admitted that families who had an orphan/abandoned child were willing to use DHM for its advantages over formula milk. They accept donated milk when a mother has lactation failure / any systemic disease / or is unfit for feeding.

The top reason to donate milk is a feeling of kindness and sisterhood, as they believe that mother’s milk has no substitute. Donating excess milk to other babies gives them happiness and satisfaction.

- ***Unwillingness for Breastmilk Donation and Utilisation***

Some mothers were unwilling to accept the donated milk for reasons such as the risk of infection to their child, loss of affection for the child, safety concerns, and loss of milk nutrients. Many mothers agreed that formula milk is more easily available than donated milk.

Similarly, the lack of knowledge and motivation, along with the cumbersome donation process, led to an unwillingness to donate milk. Travel distance also decreases the willingness to donate milk. Health care providers agreed that family customs and beliefs play a serious role in donating or utilising milk.

### 10 Practices about DHM and Mother Milk Banking

- ***Barriers to becoming donor or recipient***

Most mothers found it difficult to depart from their daily chores to come to donate. Few mothers stated expressing milk as a tedious task due to long sitting hours. Healthcare providers believe that mothers don’t have anyone to take care of their baby while she donates, thus avoid donating.

- ***Influencers in becoming donor or recipient***

Support from community leaders, social workers, Accredited Social Health activists (ASHA), Auxiliary Nurse Midwives (ANM), and Anganwadi workers can be promising to boost knowledge levels about milk banking. Together with this, the role of a lactation counselor in promoting breastfeeding is the key intent of a milk bank establishment. Reward or appreciation will act as a facilitator, as stated by one of the healthcare providers.

### B Community-related challenges

- ***Lack of Awareness and Motivation***

Mothers commented that the health education they received during pregnancy influenced their feeding choices. Thus, the role of health professionals is pivotal in educating mothers on various feeding choices and creating awareness on milk banking practices. Counseling is currently provided in groups. Some mothers required timely assistance to help overcome situations like difficulties in positioning the babies or pain during breastfeeding or while expressing milk. It also elucidates the necessity for policies to be available as written and visual information in the neonatal unit within the language(s) well understood by families and clinical staff. Milk bank managers agreed that there was a lack of community participation. Health care providers believed that myths prevailing in the community should be curbed by media and campaigns.

- ***Cultural myths and taboos***

Most healthcare providers believe that lack of information and misconceptions are major hindrances for mother milk banking. Society taboos act as a barrier for mothers, preventing them from donating or becoming a recipient. Thus, the obstacles to the acceptability of donor milk were mainly stemming from a scarcity of awareness together with the milk banking practices; these could be readily addressed through health education.

## Discussion

The Government of India is progressing to scale up the setup of the Comprehensive Lactation Management Centers (CLMC) model at the Tertiary level of public institutions with a high load of sick newborns with NICU facilities [7]. Our study provides an outline of the challenges of established mother milk banks, together with barriers and facilitators in milk bank practicing. Also, the study highlights the extent of knowledge and awareness among key stakeholders about DHM and its benefits. It also brings interesting concerns of mothers in donating or utilising milk.

The study offers insight into the present status of established AMMB in Rajasthan. Challenges such as shortage of funds, human resources, laboratory testing together with equipment maintenance, and risk of milk contamination, especially during thawing, must be addressed by ensuring adequate safety measures. Sometimes issues such as positive culture led to discarding a full batch of pooled milk. Our study emphasises the necessity to strengthen the quality and process of milk banking with technological innovation.

It was important to explore the mother’s perception of different feeding choices for the newborn. Most mothers believed that breastfeeding was superior to formula feeding. Mother Own Milk (MOM) is beneficial for child growth and also helps develop bonding. Formula feeds are costly, and moreover are likely to cause indigestion [5]. Mothers commented that the health education they received during pregnancy influenced their feeding choices. This indicates the requirement of counseling mothers during the pregnancy period and builds concepts on infant feeding based on scientific evidence for newborn development. Furthermore, family members and community leaders should also be targeted for Social Behavioral Communication Change (SBCC) interventions due to their cultural and religious influences.

In many developing countries, the use of donor human milk isn’t generally accepted [12]. In our study area, some mothers would hesitate to give donated human milk to their babies due to the risk of disease transmission; this is similar to findings in Ethiopia [13]. Maternal protective instinct decreases their willingness to utilise DHM, while the thought of sharing as an act of sisterhood gives them happiness and motivates them to donate milk. Most mothers knew the importance of breastfeeding to a child. But in conditions when the mothers have difficulty in expressing sufficient milk, the provision of DHM relieves them. Few mothers shared that donating milk is time-consuming, which acts as a barrier to their willingness to donate. Similarly, Mackenzie et al. in South Australia recorded that lactating women supported donating their expressed milk while mothers will use donated milk if safety concerns are given attention [14]. Very few mothers were unaware of milk banking. Acceptance of MMB would increase in a similar pattern as with blood donation services as mentioned by the author in one study [15].

In our study, the main reason for the unwillingness to donate breast milk and accept donated DHM for feeding was religious practices. Sociocultural values play a prominent role in influencing mother behavior. But counseling mothers on breastfeeding benefits during their pregnancy, and a further visit to NICU, help in developing faith in milk banking practices. In contrast, a study was conducted in Brazil where following NICU visits there was a 90% reduction in milk donation by mothers [16].

In *Chittorgarh AMMB*, the highest number of lactating women were registered for milk donation. This demonstrates better promotion activities, awareness, and willingness of mothers to breastfeed their children in this locale. Most of the donor mothers (70%) were in the age group of 20–32 years. The reason behind their motivation is the social value attached to breastfeeding, which is inculcated in them from a very young age.

In *Tonk AMMB*, the main barrier in counseling mothers was the cultural reservations considering milk banking as malpractice. This finding contradicts a review in Mumbai, where there was no role of specific communities in milk donation rates [15]. Also, most mothers undergo cesarean section with transfusion, which disqualifies them as per donor criteria, and they have reduced breastfeeding success [16].

In *Alwar AMMB*, the need for support from family members in the form of taking care of elder siblings, helping in household chores, and caring for the mother emerged as important findings.

Dissemination of information on milk donation within the community will help satisfy the milk requirement of babies within the government hospital along with babies who will be delivered at home or in private facilities. A study in the Metropolitan cities in India made similar suggestions: that the advantages and processes of MMBs should be shared in the community to generate awareness and acceptance for MMBs. Having an adequate number of lactation counselors, strengthening lactation support, and counseling by the service providers, especially nursing staff, will improve breastfeeding and milk expression for sufficient feeding of mothers’ own babies and donation [17].

Our study highlights the role of counseling in sustaining optimal feeding practices. Counseling is currently provided in groups; personalised and one-to-one counseling is going to be simpler. There is a need to increase the number of dedicated lactation counselors as per the load at facilities and strengthen the capacities of the staff through periodic training. Also, there should be a provision for displaying Information, Education, and Communication (IEC) material in local languages in health facilities, so it is well understood by mothers and families.

This study highlighted the role of facilitators in determining breastfeeding practices. In our study, the role of feedback from the recipient mother was also important in the successful establishment of a human milk bank. This is in contrast with the Hong Kong study, where discouragement from healthcare professionals was observed [18]. The government-supported milk banking network has successfully demonstrated its effectiveness as a part of the breastfeeding program. Keeping in mind the Indian perspective, where the frameworks about the development of human milk banks are still maturing, our study findings provide baseline information to address the barriers to the implementation of mother milk banks in India.

## Summary

- Social Behavior Communication Change (SBCC) intervention should be focused on target groups to address barriers and prevailing myths in milk banking practices.
- MMB should become an integral part of every Neonatal Intensive Care Unit. This will be a stepping stone in creating a baby-friendly world.
- Laboratory networks should be developed inside the milk bank to strengthen the quality and process of milk banking.
- New innovative tools are needed for expressing milk hygienically, even at home, to overcome societal barriers.
- The accountability of dedicated lactational counselors must be supplemented with training modules.
- Personalised and one-to-one counseling is going to be effective.
- A communication platform should be developed for problem-solving, planning promotion strategies, and social media awareness.

## Figures and Tables

**Table:** 

*Sub-Categories*	*Verbatims*
*A. **SYSTEM CHALLENGES***	
	*“I came along with my mother-in-law. She is in her 60s*.
**Structure**	*There is no sitting arrangement for her.” (M)*
	*“Sometimes we try to manage activities at our own cost*.
	*There is always a shortage of recurring funds.” (H)*
**Financial Support**	*“There should be the provision of the separate pool to protect established milk banks through constant funding.” (H)*
	*“Mothers often fail to maintain hygiene which leads to contamination of the donated milk.” (H)*
	*“Every drop of milk is precious for a sick infant. We*
**Operational process**	*screened all mothers and keep all hygienic practice throughout the process.” (H)*
	*“Mothers post-pregnancy are weak and lethargic. Long waiting period makes them tired. They should be provided some refreshment.” (H)*
	*“During COVID times, families fear the risk of transmission of the virus. We were helpless in lack of any Disaster Management plan and frequently changing guidelines.” (H)*
**Technical Process**	*“The laboratory should be functional inside mother milk bank with strong monitoring framework to ensure safety and avoid contamination.” (H)*
	*“We filed complaints a couple of times for improper functioning of equipment, but nobody replies.” (H)*
**Quality Maintenance**	*“Systematic database management is lacking.” (H)*
	*“There is a lack of systematic communication channel for problem-solving.” (H)*
**Human Resources**	*“It takes few minutes to counsel mother and families about benefits of donated human milk over formula feed. But there is the shortage of staff.” (H)*
	*“To run mother milk bank efficiently, there is need of dedicated staff.” (H)*
**Demand-Supply Ratio**	*“Donated human milk is a blessing for the child with no mother. We promote and encourage the mother to donate extra milk for orphans and abandoned child.” (H) “Motivating mothers to return for a donation once they leave hospital premises is challenging.” (H)*
	*“I lost my baby. I want to donate milk to help other mothers with less milk, feed her baby properly. It is satisfying and provides peace.” (M)*
**Knowledge about DHM & MMB**	*“Donating milk is an act of kindness. It will not only help me express more milk but will also help someone else’s baby to get food.” (M)*
	*“Expressed milk should be discarded after few hours as it loses its properties.” (H)*
	*“We promote exclusive breastfeeding as this keeps child thriving and away from infection.” (H)*
	*“If I’m convinced that donating mothers are strictly screened…I don’t have any issue using donated milk for my baby.” (M)*
	*“If no judgment is passed, I will be happy to assist a mother feed her baby.” (M)*
**Attitude (Willingness /Unwillingness to**	*“My milk is only for my child. It symbolizes my love for my child.” (M)*
**donate /utilize DHM)**	*“It sounds insane. How can I feed my child with someone else’s milk of other caste or no cultural values? What if my child inherits their values?” (M)*
	“*I wish to donate, but fear my in-laws. They don’t allow me, saying that first feed own child properly instead of donating.” (M)*
	*“My relative donated when she was lactating. She motivated me to do so.” (M)*
	*“One family directly came to us enquiring for donated milk*.
	*We counseled them and gave lactational support to the mother and it had been successful. One should understand*
**Practices (Barriers/ Influencers)**	*DHM isn’t a replacement of breastfeeding” (H)*
	*“Some communities are rigid. They treat milk banking practices as sin. We need to educate them” (H)*
	*“Some mothers hide their breastfeeding problems. Fear of in-laws is a major contributing factor to this behavior.” (H)*
***B. COMMUNITY-RELATED CHALLENGES***	
	“*In our culture, feeding our child with someone else’s milk is a bigger obligation. We can’t bear this loan” (M)*
**Awareness/Motivation**	*“Donated milk is not safe. It is a combination of milk of various mothers. Who knows if any one of them is infected?*
	*We can’t take chance with our son.” (M) “Public leaders must take this agenda in community and create awareness” (H)*
